# Acute and Subchronic Oral Toxicity of Oil Palm Puree in Sprague–Dawley Rats

**DOI:** 10.3390/ijerph17103404

**Published:** 2020-05-13

**Authors:** Zaida Zainal, Augustine Ong, Choo Yuen May, Sui Kiat Chang, Afiqah Abdul Rahim, Huzwah Khaza’ai

**Affiliations:** 1 Nutrition Unit, Product Development and Advisory Services Division, Malaysian Palm Oil Board, Bandar Baru Bangi, Selangor 43000, Malaysia; afiqah@mpob.gov.my; 2 MOSTA, C3A-10, 4th Floor, Damansara Intan, No. 1, Jalan SS20/27, Selangor 47400, Malaysia; tansriong@yahoo.com (A.O.); chooyuenmay2016@gmail.com (C.Y.M.); 3 Key Laboratory of Plant Resources Conservation and Sustainable Utilization, Key Laboratory of Post-Harvest Handling of Fruits, Ministry of Agriculture, South China Botanical Garden, Chinese Academy of Sciences, Guangzhou 510650, China; suikiatchang@gmail.com; 4 Department of Biomedical Sciences, Faculty of Medicine and Health Sciences, Universiti Putra Malaysia, Selangor 43400, Malaysia; huzwah@upm.edu.my

**Keywords:** palm puree, acute toxicity, subchronic toxicity, no-observed-adverse-effect level (NOAEL), histopathology, lethal dose 50 (LD_50_)

## Abstract

Palm puree is rich in antioxidants and is produced via blending various proportions of mesocarp fibre and crude palm oil. The aim of this study was to assess the acute and subchronic toxicity of palm puree in male and female Sprague–Dawley rats. For the acute toxicity study, animals administered single palm-puree doses (2000 mg kg^−1^) by gavage were observed daily for 14 d. For the subchronic toxicity study, the rats were administered 500, 1000, or 2000 mg kg^−1^ palm puree daily for 28 d. We evaluated body and organ weights; performed haematological, biochemical, and histopathological analyses of blood and organ samples during and after treatment; and calculated the oral no-observed-adverse-effect level (NOAEL). The toxicity studies showed no signs of toxicity or mortality. The haematological, biochemical, and histopathological analyses and body and organ weights indicated no evidence of substantial toxicity at any dose of palm puree. The oral lethal dose and NOAEL for the palm puree were greater than 2000 mg kg^−1^ d^−1^ over 28 d. To the best of our knowledge, the present study is the first to confirm the safety of palm puree as a novel functional food. These encouraging results warrant further studies to elucidate its potential for pharmaceutical formulations.

## 1. Introduction

Malaysia is a major global producer and exporter of palm oil and palm oil products [1]. The red/orange puree derived from oil palm fruit has been recognised as a functional food source [2]. Crude palm oil is extracted from the palm fruit mesocarp, and the fibre remaining after pressing is used as livestock feed. It is rich in lignin [3]. Palm puree is produced by blending various proportions of mesocarp fibre and crude palm oil (CPO). This novel product is currently being produced by the Malaysian Palm Oil Board (MPOB) and was patented by them as well (No. PI20083758) [4]. Palm puree production has helped mitigate greenhouse gas emissions by palm oil factories [5].

Palm puree is abundant in carbohydrates, proteins, and antioxidants such as carotenoids, tocols, (tocopherols and tocotrienols), phenolic acids, polyphenols, and flavonoids [3,6]. These antioxidants prolong the shelf life by enhancing the oxidative stability of palm oil and its products. Moreover, antioxidants defend cells against free radicals, which may help to prevent heart disease and cancer, and immunological, neoplastic, and neurodegenerative disorders [7]. Carotenoids are one of the groups of antioxidants present in palm puree that give an orange colour, and alpha (α)-carotene (36%) and β-carotene (54%) are the major carotenoids [8]. The tocotrienols in palm puree help to detoxify certain products generated by aerobic metabolism [9]. Palm puree production complies with safety specifications for the preparation and processing of foods destined for human consumption. The palm puree fabrication process has been certified under Hazard Analysis and Critical Control Points (HACCP) [5,6]. The increased demand for palm oil has created a multiplier impact on the tropical conservation forests, destroying the biodiversity of some ecosystems and leading to the degradation of habitats of species, triggering social tensions between indigenous communities and farmers. Thus, Malaysia has launched its own voluntary Malaysian Sustainable Palm Oil (MSPO) certification programme to address concerns among growers [10]. 

Compounds must be confirmed as nontoxic to humans before they are authorised for use as prescription drugs or nutraceuticals [11]. To the best of our knowledge, the present study is the first to execute a systematic investigation into the potential toxicity of palm puree before it can be integrated into functional food and nutraceutical formulations and validated for safety and efficacy in human clinical trials. The present acute and subchronic toxicity assessments may help clarify the animal exposure limits and safety thresholds for palm puree and predict the exposure levels that could possibly be harmful to humans. Here, the impact of concentrated palm puree on numerous biochemical, haematological, and histopathological parameters in rats were investigated in the aforementioned acute and subchronic toxicity assays. 

## 2. Materials and Methods

### 2.1. Preparation of Palm Puree Extract

Palm puree was extracted from oil palm mesocarp fibre and collected at the Taitak Palm Oil Mill in Johor, Malaysia. First, crude palm oil was extracted from fresh fruit bunches (FFB) after washing the palm fruits with water. Then, the cleaned fruits were subjected to the conventional palm oil milling process. The low oil food source or palm puree was obtained from the aqueous stream of the process. The puree was sprayed with nitrogen to prevent oxidation then stored in an airtight container at 5 °C until further investigation. It was dissolved in water and filtered before use. The OECD (Organization for Economic Cooperation and Development) guidelines state that the palm puree dosage (mg) must be constituted in a volume ≤ 10 mL kg^−1^ (1 mL 100 g^−1^) body weight (BW) for the investigation of non-aqueous solvents in treated rats by oral gavage [12]. The palm puree was formulated as water suspensions with concentrations of 55, 110, and 220 mg mL^−1^ corresponding to the lower (500 mg kg^−1^ BW), moderate (1000 mg kg^−1^ BW), and high doses (2000 mg kg^−1^ BW), respectively, where ≤ 2 mL of each was administered to each rat. The nutritional composition of palm puree water extract is shown in Table 1. 

### 2.2. Experimental Animals

Healthy male and female specific pathogen-free Sprague–Dawley (SD) rats aged 8 weeks (male: 200–220 g; female: 160–190 g) were used in both the acute and subchronic toxicity studies. The rats (*n* = 80) were obtained from Sterling Sdn. Bhd., Kuala Lumpur, Malaysia and acclimatised to laboratory conditions for 14 d before the experiments. Three male or three female rats were placed in different cages and fed a normal diet and altromin (Altromin 1324 FORTI; Altromin, Lage, Germany) fortified with 25,000 IU of vitamin A, 1000 IU of vitamin D3 (cholecalciferol), and 125 mg of vitamin E (tocopherol). The diet composition was 7.5% crude ash, 6.1% crude fibre, 4.1% crude fat, 19.2% crude protein, 53.4% free nitrogen, and 11.3% moisture. The rats were kept in a room at 23 ± 3 °C and 55% ± 15% relative humidity (RH), with artificial lighting (12 h; 08:00–20:00 h) and provided with ultrasterile water ad libitum. Each cage was labelled with the unique identification numbers of each animal.

### 2.3. Study Design

The number of rats used and the experimental procedures received endorsement from the Institutional Animal Care and Use Committee (IACUC) of Universiti Putra Malaysia, Serdang, Selangor, Malaysia and strictly complied with international standard animal laboratory principles. Ethical approval was granted on 2nd June 2014 (No. UPM/IACUC/AUP-R042/2014) by IACUC/101.

### 2.4. Acute Oral Toxicity Study

The toxicological properties of palm puree were examined according to the OECD Guideline No. 423 in a single-dose, 14 d acute oral toxicity study. Three animals of the same sex were used per treatment. Five male and five female rats were fasted overnight before treatment and then orally administered 2000 mg kg^−1^ palm puree extract in a single dose. The rats in the control group were administered a standard diet without palm puree. The rats were closely observed in terms of their general behaviour, adverse clinical signs, and mortality for the first hour and subsequently for 3 h at regular intervals for 48 h. The rats were then euthanised by injectable anaesthetic solution (ketamine:xylazine) until all the rats were unconscious, following which blood samples were drawn by cardiac puncture for haematological and biochemical investigations. Next, internal organs such as the heart, kidney, lung, liver, and spleen were excised, weighed, and macroscopically inspected according to OECD Guideline No. 423 [13].

### 2.5. Subchronic Oral Toxicity Study

The subchronic oral toxicity of palm puree was assessed according to OECD Guideline No. 407 [14]. Approximately 80 healthy SD rats of both sexes were weighed and randomly separated into three test groups and a control group (*n* = 10; five males + five females) as follows: Group 1 (control), Group 2 (500 mg kg^−1^ BW), Group 3 (1000 mg kg^−1^ BW), and Group 4 (2000 mg kg^−1^ BW).

A standard palm puree formulation was dissolved in distilled water and orally administered to rats daily for 28 d at single doses of 500 mg kg^−1^, 1000 mg kg^−1^, and 2000 mg kg^−1^. The control group received only distilled water. All rats were closely monitored for behavioural changes, mortality, toxic manifestations, BW alterations, nutrient consumption and assimilation, and clinical signs for 28 d. BW was measured weekly. At the end of the experiment (Day 28), all rats were fasted overnight, and blood samples were drawn by cardiac puncture under 50 mg kg^−1^ BW ketamine anaesthesia. The rats were then euthanised by clavicle dislocation; the livers, kidneys, brains, spleens, lungs, adrenal glands, and hearts were excised, weighed, and macroscopically examined. All organs were fixed in 10% (v/v) formalin for histopathological analysis.

### 2.6. Blood Analyses

Blood samples were drawn by cardiac puncture for haematological and biochemical investigations. They were transferred to heparinised tubes containing ethylenediaminetetraacetic acid (EDTA) for the evaluation of haematological parameters and to non-heparinised tubes for biochemical examinations. The tubes were centrifuged at 3000× *g* at 4 °C for 10 min to separate the sera, which were then stored at −20 °C until subsequent analyses.

### 2.7. Haematological Assay

The blood samples in the heparinised tubes were used for haematological analyses including white blood cell count (WBC), red blood cell count (RBC), mean corpuscular haemoglobin (MCH), mean corpuscular haemoglobin concentration (MCHC), packed cell volume (PCV), and neutrophil, lymphocyte, and differential leucocyte numbers. The aforementioned measurements were made with a total autoanalyser/automated biochemistry analyser (HITACHI 902 Automatic Analyzer^®^; Hitachi, Tokyo, Japan).

### 2.8. Biochemical Analyses

Sera were collected to measure potassium (K), sodium (Na), cholesterol (CHO), glucose (GLU), triglycerides (TG), calcium (Ca), chloride (Cl), creatinine (Crea), alanine aminotransferase (ALT), aspartate aminotransferase (AST), alkaline phosphatase (ALP), total protein (TPRO), creatinine kinase (CK), phosphorus (P), urea, lactate dehydrogenase (LDH), and albumin (ALB) using a COBAS Integra 800 (Roche Diagnostics, Basel, Switzerland) assay kit according to the manufacturer’s instructions.

### 2.9. Necropsy and Histopathological Examination

All rats were inspected for internal and external injury. The necropsy included inspection of the outer surfaces, the orifices, and the thoracic and gastrointestinal cavities. The hearts, livers, lungs, kidneys, and spleens were excised and weighed. The relative organ weights were calculated as follows [15]:

(weight of internal organ/final BW of the rat) × 100(1)

Histopathological assessments were conducted on the kidneys, livers, lungs, hearts, and spleens. The vital organs were maintained in 10% (v/v) formalin. The organs of interest were trimmed, embedded in paraffin, and sectioned into slices of 5 µm thickness. The tissue sections were stained by haematoxylin and eosin and inspected under a light microscope for histopathological changes. Some or all of the following were observed in the lungs: perivascular/vascular and luminal vacuolation, granulomatous inflammation, perivascular mononuclear cell infiltration, foamy macrophage accumulation, and tunica media thickening in the small- and mid-sized blood vessels.

### 2.10. Statistical Analysis

All data were quantified as the means ± standard error of the mean (SEM) in triplicate independent analyses. Experimental means were analysed for significance using paired samples t-tests and one way analysis of variance (ANOVA) followed by Bonferroni’s test as a post hoc test to identify differences among treatment means for all biochemical and haematological parameters and body and organ weights. *p* < 0.05 was the threshold for statistical significance.

## 3. Results

### 3.1. Acute Toxicity Study in Rats

#### 3.1.1. Mortality

Oral palm puree (2000 mg kg^−1^) did not induce any abnormal responses in either the treatment or control groups over the 14 d observation period. All rats presented the same appearance and behaviour throughout the observation period. There were no mortalities in any of the groups during the study period.

#### 3.1.2. Body Weight

**Figure 1:**
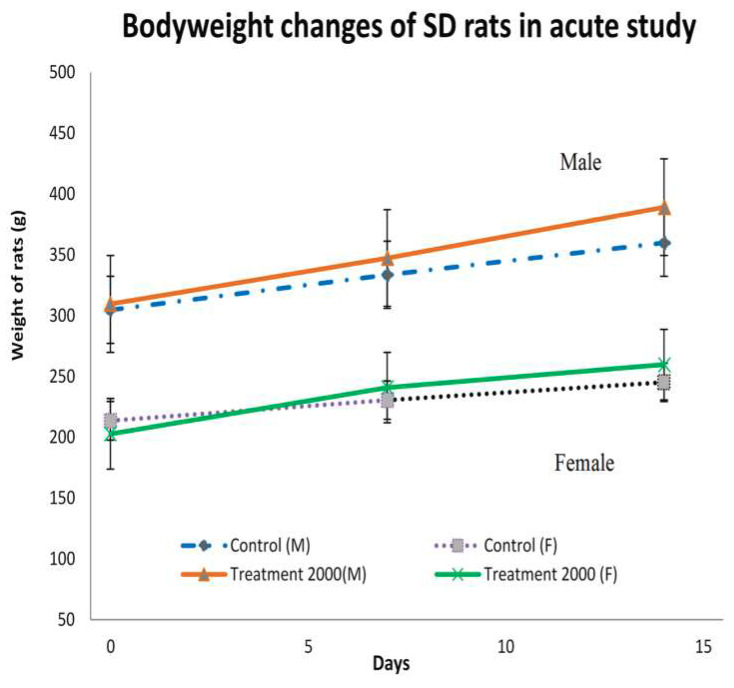
Average body weight gain for male (A) and female (B) rats treated by gavage with 0 mg kg^−1^ (◊) and 2000 mg kg^−1^ (x) palm puree for 14 d during an acute toxicology study (*n* = 6 animals per group). Data are means ± SD for groups of five animals/sex/dose.

The body weight (BW) gain rates were normal for all rats (Figure 1). However, the weight gain differential before and after treatment was significant.

#### 3.1.3. Effects of Palm Puree on Relative Heart, Liver, Kidney, Lung, and Spleen Weights

Table 2 shows that the intact heart, liver, kidney, spleen, and lung weights were transformed relative to 100 g BW. The administration of palm puree for 14 d had no significant effect on organ weight. There was no significant difference (*p* > 0.05) between the control and treatment groups in terms of relative organ weight. Pathological examinations of the hearts, livers, kidneys, lungs, and spleens of the treated rats revealed no significant differences in shape, size, texture, or colour compared to those of the control rats. These findings suggest that the palm puree is nontoxic.

#### 3.1.4. Effects of Oral Palm Puree Administration on Rat Haematological Parameters in Acute Toxicology Study

The acute toxicity data (Table 3) revealed that, compared with the control group, the rats treated with 2000 mg kg^−1^ palm puree did not show a significant difference (*p* > 0.05) with respect to haematological parameters, including red blood cell count (RBC); packed cell volume (PCV); mean corpuscular volume (MCV); mean corpuscular haemoglobin (MCH); platelet number; and neutrophil, lymphocyte, monocyte, and eosinophil counts. A significant increase in haemoglobin (*p* < 0.05) and significant decrease (*p* < 0.05) in WBC values was noted between both treated male and female rats. Certain differences were present between treated male and female rats. For example, significant decrease (*p* < 0.05) in MCHC values was noted in treated female rats. However, a non-significant increase in MCHC values was noted in treated male rats. Nevertheless, all these changes were still within the normal reference range for rats. 

#### 3.1.5. Effects of Oral Palm Puree Treatment on Rat Biochemical Parameters

Table 4 shows significant changes (*p* < 0.05) in the triglyceride levels of treated male rats compared to those for the control group. By contrast, there were only slight variations in the triglyceride levels of the treated female rats relative to the control. The cholesterol levels of the treated female rats did not substantially differ from those for the control. However, the cholesterol levels of the treated male rats differed substantially from those for the control. LDH and CK were significantly lower (*p* < 0.05) in both the treated male and female rats than in the control (Table 4).

There were no significant differences between the treated and control male and female rats in terms of any biochemical parameter except the sodium, calcium, glucose, uric acid, and AST levels in the treated male rats. The latter animals presented with significant relative reductions in AST (*p* < 0.05 at 2000 mg kg^−1^) and glucose (*p* < 0.01 at 2000 mg kg^−1^) relative to the control. On the other hand, significant (*p* < 0.05) increases in ALT and AST levels and marked decreases in ALP and uric acid levels were observed in the treated female rats (Table 4). TG and cholesterol were reduced. CK was reduced by half. Uric acid was reduced by half. 

### 3.2. Subchronic Toxicity Study

#### 3.2.1. Effects of Subchronic Oral Palm Puree Treatment on Rat Body and Organ Weights

**Figure 2:**
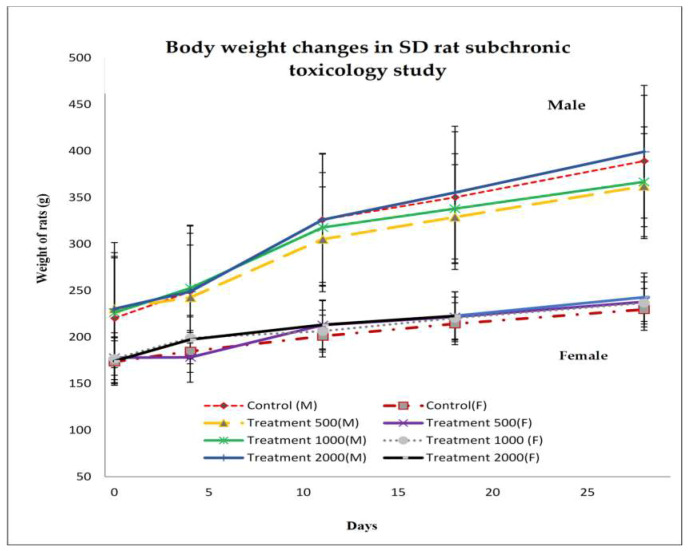
Average body weight gain for male (A) and female (B) rats administrated palm puree at 0 mg kg^−1^ (◊), 500 mg kg^−1^ (∆), 1000 mg kg^−1^ (●), and 2000 mg kg^−1^ (│) by oral gavage during a 28 d subchronic toxicity study (*n* = 5 animals per group). Data are means ± SD for each group. Data are means ± SD for groups of five animals/sex/dose.

Mean BWs did not significantly differ (*p* > 0.05) between the treated and control rats. In fact, they were nearly equal. All animals presented with reasonable BW increments without any drastic difference between the control and treated groups. Progressive increases in BW were recorded for both the male and female rates at 500 mg kg^−1^, 1000 mg kg^−1^, and 2000 mg kg^−1^ over 28 d of palm puree administration (Figure 2). Thus, the nutritional status of the animals had improved over time with these treatments. Subchronic oral palm puree administration induced no observable changes in the general behaviours of the treated rats compared to those of the controls. The treated rats manifested no visible changes in the skin, fur, ocular mucous membranes, or behavioural patterns. They presented with no apparent tremors, salivation, or diarrhoea. Both the control and treated rats were healthy throughout the entire 28 d treatment period. No death resulted from the rats being fed ≤ 2000 mg kg^−1^.

Table 5 shows the relative organ weights of male and female rats after 28 d palm puree administration. Overall, there were no significant differences among the relative organ weights. However, the principal organ weights like male lung weight at 1000 and 2000 mg/kg significantly increased (*p* < 0.05) in the treated rats relative to the control. Similarly, the relative female rat lung weights were significantly greater for the 500, 1000, and 2000 mg kg^−1^ treatment groups than the control (*p* < 0.05). We observed that compared to the control, there were slight increments in male liver weight at the 1000 mg kg^−1^ and 2000 mg kg^−1^ palm puree doses. Nevertheless, these differences were not significant (*p* > 0.05). The treated female rats presented with higher liver, spleen, and lung weights than the male rats.

#### 3.2.2. Effects of Subchronic Oral Palm Puree Administration on Rat Haematological Parameters

There were no significant differences (*p* > 0.05) between the treated and control male and female rats in terms of RBC, haemoglobin, PCV, MCV, or WBC (Table 6). The neutrophil counts in the male rats treated with 2000 mg kg^−1^ palm puree were significantly lower (*p* < 0.05) than those in the control and those administered 500 mg kg^-1^ or 1000 mg kg^−1^ palm puree daily. The lymphocyte counts of the male rats fed 1000 mg kg^−1^ and 2000 mg kg^−1^ were significantly lower (*p* < 0.05) than those of the control.

#### 3.2.3. Effects of Subchronic Oral Palm Puree Administration on Rat Biochemical Parameters

The impacts of subchronic oral palm puree administration on the biochemical parameters are presented in Table 7. None of the treatments had any substantial influence on these factors. No significant changes in the lipid profile except for TG were observed in the treated male and female rats. Relative to the control, the TG was markedly higher for the male rats administered 2000 mg kg^−1^ palm puree. However, the value was still within the normal range. By contrast, the female rats administered 2000 mg kg^−1^ palm puree presented with significantly reduced TG compared to that of the controls.

Palm puree slightly reduced the serum electrolytes (sodium, potassium, and chloride) relative to the untreated control but the differences were not significant (*p* > 0.05). The levels of urea and creatinine (kidney function biomarkers) did not change in response to palm puree administration relative to the control. However, uric acid levels were significantly reduced (*p* < 0.05) in male rats administered 2000 mg kg^−1^ palm puree compared to those in the controls. Overall, the kidney performance parameters (urea, creatinine, and acid) did not substantially change in response to palm puree administration. By contrast, uric acid levels were slightly elevated in treated female rats at 2000 mg kg^−1^ relative to levels in the controls, but the difference was not significant (*p* > 0.05).

### 3.3. Histopathological Study

**Figure 3:**
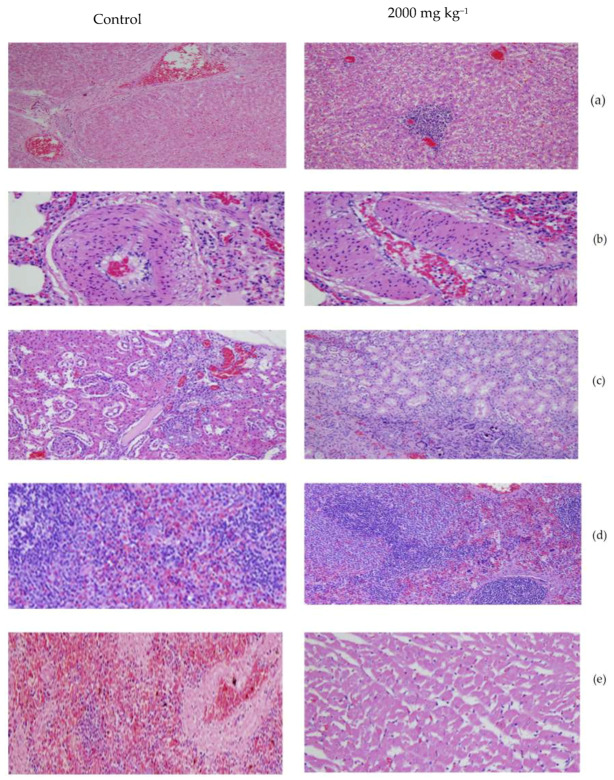
Histological examinations of liver (**a**), lung (**b**), kidney (**c**), spleen (**d**), and heart (**e**) (schedule in 200 µm) from representative male rats treated with vehicle (control) or palm puree. Effects of 2000 mg kg^−1^ palm puree on various organs in a subchronic oral toxicity study. Top: organ (200×) and respective control group shown.

Hematoxylin and eosin stain (H&E)-stained rat liver, heart, kidney, lung, and spleen tissue sections were histopathologically examined. On a microscopic level, the lungs of all treated rats presented with some or all of the following pulmonary anomalies: perivascular/vascular and luminal vacuolation, granulomatous inflammation, perivascular mononuclear cell infiltration, foamy macrophage accumulation, and tunica media thickening in both small and mid-sized blood vessels (Figure 3). The other microscopic findings observed in all animals (treated and control) were considered incidental or spontaneous and not associated with any toxic effect of palm puree.

On a microscopic level, no palm puree-related changes were observed in any of the aforementioned organs. In a rat treated with 2000 mg kg^−1^ palm puree, we observed multifocal moderate granulomatous inflammation and a foreign body in the lungs. This response could be attributed to the aspiration of gastric reflux. All other microscopic findings noted in control and treated animals were considered incidental or spontaneous and not due to the toxicological effect of palm puree. 

## 4. Discussion

The aims of the present study were to evaluate possible toxic effects of palm puree administration in rats and, by extension, to assess whether this food could cause harm to humans. Olson et al. (2000) [16] reported that unfavourable haematological, gastrointestinal, and cardiovascular effects observed in animals have the highest concordance with those in humans. By contrast, hypersensitivity and idiosyncratic reactions in humans are poorly correlated with toxicity symptoms in animals.

Here, it was observed that some rats presented with BW gain in both the acute and subchronic palm puree toxicity assays. Changes in rat body and internal organ weights after exposure to putatively toxic substances are indicative of toxicity [17]. Alterations in body and organ weights were observed after rats were administered with 2000 mg kg^−1^ palm puree [18]. A loss of >10% of the preliminary weight may indicate severe toxicity [19].

BW had significantly increased in both female and male rats within 8 d after oral palm puree administration in the acute toxicity study. According to Teo et al. (2002) [20], the probability of exposure to potentially toxic substances was low if no significant differences in weight gain between control and treated rats are detected. Raza et al. (2002) [21] stated that BW reduction could indicate side effects or adverse reactions to substances. In the present acute toxicity study, the rats were administered a single dose (2000 mg kg^−1^) of palm puree that induced progressive weight gain significantly differing (*p* < 0.05) from that observed in the control. No death or sign of toxicity was noted for any group at 14 or 28 d after palm puree administration. Therefore, palm puree produces no toxic effects at doses ≤ 2000 mg kg^−1^ BW, indicating that it is essentially nontoxic.

The acute and subchronic palm puree toxicity studies on treated and control rats disclosed critical weight variation among treatments. All rats exhibited daily body increments, but there were no substantial differences in these rates between the control and treatment groups. A plausible explanation is that the palm puree administration improved the nutritional status in the rats. All treated and control rats were fit and healthy during the 28 d experimental period. No death or sign of toxicity was recorded for any of the animals after 28 d. These outcomes confirm that palm puree is essentially nontoxic at doses ≤ 2000 mg kg^−1^ BW.

Another index of the potential toxicity of a substance is the effect it might have on relative organ weights [19]. The heart, liver, kidneys, spleen, and lungs are the first organs to display metabolic responses to poisonous substances [22]. Thus, relative organ weight scould serve to diagnose any possible injury to organs exposed with toxic materials. Normally, the consumption of toxic substances harms bound organs [20]. Organ weight is a highly sensitive indicator of drug toxicity. Important differences in organ weight between treated and control animals may occur without any morphological changes in response to toxicant exposures [23]. In the acute palm puree toxicity study, no significant differences (*p* > 0.05) were detected between the treatment and control rats of both sexes in terms of relative spleen, liver, kidney, or lung weights. There were also no variations among groups in terms of the gross appearance, size, colour, or microscopic characteristics of the internal organs. However, the lung will be the subject of more detailed experiments.

In the subchronic toxicity study, however, there were significant differences (*p* < 0.05) between the control and the male rats administered 2000 mg kg^−1^ palm puree and between the control and the female rats administered all three palm puree doses in terms of microscopic lung appearance and structure. Piao et al. (2013) [24] reported that changes in lung weight are not associated with any toxicologic effect of oral drug or substance administration. Moreover, the measured lung weights here were within the normal range for all animals. In the present study, some the relative organ weights of the rats treated with palm puree were markedly greater than those of the control. Thus, palm puree may confer some protective effect on the organs. As there were no reductions in body or relative organ weights in any of the treated rats, palm puree was basically nontoxic to all organs analysed here.

The haematopoietic system is highly sensitive to toxicants. Haematological studies are vital indices of the pathophysiological status of animals and humans [25]. A significant increase in haemoglobin (*p* < 0.05) and significant decrease (*p* < 0.05) in WBC values was noted between both treated male and female rats after 14 d. In addition, certain differences were present between males and female rats. For example, a significant decrease (*p* < 0.05) in MCHC values was noted in treated female rats. However, a non-significant increase in MCHC values was noted in treated male rats after 14 d. These results indicate biological sex-based differences, which warrant further investigation. There were no significant differences (*p* > 0.05) in any haematologic parameter between rats of both sexes treated with palm puree extract and the control after 28 d. The palm puree had no apparent effect on rat haemogenesis or leucopoiesis. In haematopoiesis, cellular blood elements are synthesised whereas in leucopoiesis, WBCs are synthesised in adult bone marrow and foetal haemopoietic organs [26].

The RBC and WBC values slightly decreased in both female and male rats in a dose-dependent manner relative to the control. The RBCs contain haemoprotein that carries oxygen, and RBC assays can diagnose anaemia and other related conditions. Free phagocyte counts may quantify infection-fighting WBCs in the blood. WBCs comprise basophils, eosinophils, lymphocytes, monocytes, and neutrophils, and their numbers increase in response to infections and allergic reactions. High WBC counts may be associated with cancers, microbial infections, and other diseases [27]. 

The neutrophil counts in male rats treated with 2000 mg kg^−1^ palm puree were slightly but not significantly (*p* > 0.05) lower than those in the control as were those in male rats administered 500 mg kg^−1^ or 1000 mg kg^−1^ palm puree. Neutrophils defend the body against infections whereas lymphocytes are major effectors of the immune system [28]. All rats treated with palm puree presented with slightly lower lymphocyte counts than the control, but the values were still within the normal range. Thus, palm puree does not substantially challenge the rat immune systems. Li et al. (2006) [29] reported that rats administered graphene had leucocyte densities in the range of 3.7–5.5 (10^9^ L^−1^). Here, the acute and subchronic toxicity studies over 14 d and 28 d demonstrated that daily oral palm puree administration was not necessarily harmful to the rat blood system.

Biochemical analyses were conducted to establish whether palm puree could alter hepatic or renal function in rats. Liver and kidney functional analyses are indices of drug and plant extract toxicity [30]. The liver metabolises potentially toxic “foreign materials” that have been ingested [31]. The results showed that the TG and cholesterol values were reduced significantly in treated male rats while the creatine kinase (CK) and uric acid values demonstrated significant reductions in both treated male and female rats after supplementation with palm puree for 14 d. These results showed that the palm puree has therapeutic benefits for lipid profiles. The upregulation of liver enzymes such as ALT, AST, and ALP indicates liver injury. ALT is closely associated with liver function whereas AST is found mainly in the myocardium, skeletal muscle, brain, and kidneys. Significant increases in the activity and serum levels of these enzymes may be related to liver damage [32].

In the present study, palm puree administration slightly but non-significantly elevated serum ALP relative to the control. Serum ALP was slightly higher in rats administered 2000 mg kg^−1^ BW for 28 d than in the control. A reduction in serum AST indicates hepatocellular enzyme production or an inhibition or reduction in enzyme activity [33]. However, AST is also found in the heart and muscles and is not specific to acute liver diseases [34]. Here, non-significant changes in ALP, ALT, and AST were observed in the male and female rats at all palm puree doses relative to the control. Thus, subchronic palm puree administration did not affect rat hepatocyte function. Unlike AST, ALT is mainly localised to the liver. The European Document for Ecotoxicology and Toxicology (2002) [35] stated that the biological significance and impact of decreases in liver enzyme activity and level are unclear. For this reason, this factor is regarded as toxicologically unimportant in the context of the present study.

Reductions in total protein, albumin, and globulin also affect liver function. As the liver is a major site of protein synthesis, any decrease in its function may be indicative of hepatocyte damage. Impaired hepatocellular function may result in reductions of serum albumin, total protein, and bilirubin concentrations [36]. Albumin prevents fluids from leaking out of blood vessels 41. Low albumin levels may also be characteristic of infection [37]. Our data revealed that no palm puree dose significantly influenced serum total protein or albumin in male or female rats. Therefore, none of the tested palm puree doses damaged hepatocellular function.

Kidney function was inferred by parallel urea, creatinine, and uric acid analyses [38]. The levels of these three biomarkers did not significantly differ between the control and any of the palm puree treatment groups. However, serum creatine kinase (CK) was dramatically lower in all treated male and female rats than in the control. This finding was consistent and correlated with the amount of protein in the blood. CK is an enzyme in the muscles and its levels rise in response to heart or muscle damage [39].

LDH is an enzyme occurring in many different types of cell and is a biomarker of tissue damage. Though the LDH levels in the treatment groups were markedly lower than those in the control, they were nonetheless still within the normal range, namely, 60–160 U L^−1^ [40]. The serum glucose levels of treated males and females were slightly higher than those of the control but still within the normal range for this parameter. According to Sprague and Arbelaez (2011), the serum glucose was sufficiently low to be considered hypoglycaemic [41]. Significantly lower sodium and chloride levels in male rats were observed during the acute oral toxicity study. However, this electrolyte imbalance apparently resolved during the chronic toxicity study. This could be due to the adaptation of the physiological system after a long duration as compared to in the acute toxicity study. Homeostasis kept the internal environment constant by correcting the electrolyte imbalance [42]. However, the significant reduction in potassium levels as observed in rats treated with 1000 and 2000 mg kg^−1^ requires a chronic toxicity study for 90 days to be carried out in the future. Overall, no major significant differences were detected between the control and the treated rats of both sexes in terms of the levels of electrolytes including sodium, potassium, and chloride. Electrolyte stability is vital for normal cell and organ functioning [42].

Histological examinations identify changes in the cell structure of internal organs not necessarily reflected by the changes detected by haematological and biochemical investigations [17]. Here, histopathological inspections after both acute and subchronic oral palm puree ingestion revealed that palm puree did not affect major rat organ morphology; the liver, heart, kidneys, lungs, and spleen showed no toxic effects even after repeated oral palm puree administration at 500, 1000, or 2000 mg kg^−1^ for 14 and 28 d. Several animals in all treatment groups presented with minimal to mild thymic haemorrhage, which may have been an agonal response to the euthanasia [43]. The control rats presented with intact hepatocytes, portal veins, glomeruli, and tubules. No gross abnormalities were observed in the organs of the animals subjected to acute and subchronic palm puree administration. Histopathological examinations of the livers and kidneys of male and female rats administered palm puree presented with no lesions or other pathological changes. All other microscopic anomalies recorded for the control and treated rats were considered incidental or spontaneous and were not associated with any toxicological effect.

## 5. Conclusions

The present study disclosed that palm puree and its constituents had no effect on rat mortality. Oral palm puree administration at doses of 500, 1000, and 2000 mg kg^−1^ d^−1^ did not induce toxicity, mortality, or pathological abnormalities in any internal organ. Palm puree caused no apparent *in vivo* toxicity in any treated rats even at the maximum tested dose of 2000 mg kg^−1^. Histopathological examinations revealed no significant alterations in the architecture of the internal organs of treated rats relative to the control. However, a further chronic toxicity study for 90 days using the three dosages needs to be conducted to ascertain the minor alterations in the lungs and the biochemical and haematological parameters affected by palm puree.

Based on the criteria in the Guidance Document on Acute Oral Toxicity Testing based on the oral lethal dose (LD_50_) value and endorsed by the Organization for Economic Cooperation and Development (OECD, 2001) [43], crude palm puree extract may be categorized as classification No. 5 (LD_50_ = 2000 mg kg^−1^), which is the lowest toxicity category. As such, no label is required. Palm puree may therefore be considered safe for use in food formulations. The oral no-observed-adverse-effect level (NOAEL) for palm puree was >2000 mg kg^−1^ d^−1^. However, as the foregoing toxicity studies were only conducted on experimental rats, the outcomes cannot and should not be extrapolated to humans. Thus, clinical toxicological research must be conducted in the future. However, the results of the current toxicity assessment of palm puree are beneficial to the crude palm oil and food industries, as well as to pharmaceutical companies. As palm puree was effectively nontoxic to rats even at the highest doses tested, this material could be tested for medicinal and therapeutic efficacy.

## Figures and Tables

**Table 1:** Nutritional composition of palm puree water extract [5].

Palm Puree Nutrition Information	
Energy	400–700 kcal 100 g^−1^
Calories	350–600 kcal 100 g^−1^
Carbohydrates	1.3%–2.8%
Proteins	0.6%–2.1%
Ash content	0.5%–1.5%
Total fats	40.5%–54.8%
Monounsaturated fats	−22.3%
Polyunsaturated fats	−5.5%
Saturated fats	1.5%–3.2%
Carotenoids	26–75 mg 100 g^−1^
Tocols	
α-Tocopherol	10–20 mg 100 g^−1^
α-Tocotrienol	8–15 mg 100 g^−1^
γ-Tocotrienol	30–58 mg 100 g^−1^
δ-Tocotrienol	7–35 mg 100 g^−1^
Vitamin B complex	ND–10 mg 100 g^−1^
Vitamin C	ND–10 mg 100 g^−1^

**Table 2:** Relative rat organ weights after treatment with palm puree during a 14 d acute toxicology study. Data are means ± SD for groups of five animals/sex/dose. * (*p* < 0.05) vs. control group.

Parameters	Dose (mg kg^−1^)	
	0	2000
Male		
Heart	0.31 ± 0.02	0.35 ± 0.02
Kidney (R)	0.33 ± 0.10	0.37 ± 0.04
Kidney (L)	0.34 ± 0.02	0.32 ± 0.02
Liver	3.81 ± 0.13	3.80 ± 0.08
Spleen	0.22 ± 0.02	0.22 ± 0.02
Lung	0.56 ± 0.03	0.57 ± 0.06
Female		
Heart	0.31 ± 0.05	0.32 ± 0.02
Kidney (R)	0.32 ± 0.07	0.33 ± 0.05
Kidney (L)	0.33 ± 0.04	0.32 ± 0.03
Liver	3.61 ± 0.06	3.80 ± 0.02 *
Spleen	0.23 ± 0.07	0.22 ± 0.02
Lung	0.55 ± 0.03	0.54 ± 0.05

**Table 3:** Haematological values for rats treated with palm puree in an acute toxicity study. Data are means ± SD for groups of five rats/sex/dose. * (*p* < 0.05) vs. control group.

Parameter	Dose (mg kg^−1^ d^−1^)	
	0	2000
Male		
RBC (× 10^12^ L^−1^)	5.07 ± 1.71	5.19 ± 1.21
Haemoglobin (g L^−1^)	131.75 ± 15.11	142.4 ± 12.48 *
PCV (L L^−1^)	0.27 ± 0.10	0.27 ± 0.06
MCV (fL)	52.33 ± 1.75	52.5 ± 2.43
MCHC (g L^−1^)	361.33 ± 25.04	365.17 ± 20.18
WBC (× 10^9^ L^−1^)	3.28 ± 2.52	2.67 ± 1.22 *
Neutrophils (× 10^9^ L^−1^)	0.27 ± 0.23	0.27 ± 0.11
Lymphocytes (× 10^9^ L^−1^)	2.83 ± 2.16	2.32 ± 1.04
Female		
RBC (× 10^12^ L^−1^)	5.45 ± 1.3	6.09 ± 1.59
Haemoglobin (g L^−1^)	132.6 ± 9.42	136.6 ± 9.50 *
PCV (L L^−1^)	0.28 ± 0.07	0.33 ± 0.09
MCV (fL)	51.5 ± 3.39	53.67 ± 4.18
MCHC (g L^−1^)	366.50 ± 19.20	348.17 ± 23.03 *
WBC (× 10^9^ L^−1^)	3.18 ± 1.18	3.00 ± 1.52 *
Neutrophils (× 10^9^ L^−1^)	0.27 ± 0.18	0.29 ± 0.20
Lymphocytes (× 10^9^ L^−1^)	2.29 ± 1.25	2.53 ± 1.21

RBC: red blood cell count, MCV: mean corpuscular volume, MCH: mean corpuscular haemoglobin, MCHC: mean corpuscular haemoglobin concentration, WBC: white blood cell count, PCV: packed cell volume.

**Table 4:** Serum biochemical data for treated rats in a 14 d acute oral toxicity study. Data are means ± SD for groups of five animals per treatment. * (*p* < 0.05) vs. control group.

Parameters	Dose (mg kg^−1^)	
	0	2000
Male		
Trig (mM)	1.12 ± 0.62	0.86 ± 0.36 *
LDL (mM)	0.18 ± 0.04	0.19 ± 0.08
HDL (mM)	0.74 ± 0.22	0.72 ± 0.27
Chol (mM)	1.38 ± 0.42	1.28 ± 0.38 *
LDH (mM)	2091 ± 16.88	1187 ± 23.8 *
Lact (mM)	3.65 ± 01.19	4.41 ± 02.00 *
CK (U L^−1^)	852.70 ± 23.5	463.3 ± 12.8 *
Total protein (g L^−1^)	40.13 ± 0.08	37.80 ± 0.57 *
Albumin (g L^−1^)	25.23 ± 0.46	24.42 ± 0.32
Na (mM)	140.45 ± 0.41	113.82 ± 0.89 *
K (mM)	6.57 ± 0.88	5.33 ± 0.61
Cl (mM)	96.12 ± 0.3	80.37 ± 0.84 *
Uric acid (µM)	120.9 ± 0.17	96.2 ± 0.74 *
Urea (mM)	5.42 ± 0.73	5.13 ± 0.25
Creatinine (mM)	35.17 ± 0.14	34.33 ± 0.66
ALT (mM)	26.8 ± 9.26	31.48 ± 0.41 *
ALP (U L^−1^)	117.33 ± 0.95	116.67 ± 0.39
AST (U L^−1^)	105.78 ± 7.19	68.48 ± 8.79 *
Glucose (mM)	12.23 ± 0.14	8.92 ± 0.74 *
Phos (mM)	1.55 ± 0.46	1.52 ± 0.34
Amylose (U L^−1^)	1581.20 ± 18.68	1694.3 ± 10.45 *
Female		
Trig (mM)	0.65 ± 0.16	0.57 ± 0.14
LDL (mM)	0.29 ± 0.09	0.26 ± 0.08
HDL (mM)	1.46 ± 0.19	1.45 ± 0.18
Chol (mM)	2.28 ± 0.32	2.09 ± 0.80
LDH (mM)	1920.33 ± 35.2	569.64 ± 74.1 *
Lact (mM)	3.07 ± 0.97	3.12 ± 0.5
CK (U L^−1^)	790.2 ± 37.7	319.2 ± 52.5 *
Total protein (g L^−1^)	57.22 ± 5.36	56.65 ± 0.75
Albumin (g L^−1^)	38.27 ± 0.72	34.13 ± 0.53
Na (mM)	138.45 ± 0.67	143.12 ± 0.12
K (mM)	6.67 ± 0.59	5.05 ± 0.39
Cl (mM)	98.18 ± 0.49	99.43 ± 0.51
Uric acid (µM)	107.33 ± 0.8	63.8 ± 0.58 *
Urea (mM)	6.43 ± 0.11	6.42 ± 0.64
Creatinine (mM)	49.33 ± 0.97	46.83 ± 0.14
ALT (mM)	37.83 ± 0.64	47.31 ± 0.75 *
ALP (U L^−1^)	117.33 ± 0.91	116.67 ± 2.51
AST (U L^−1^)	105.78 ± 14.5	97.85 ± 21.4 *
Glucose (mM)	13.52 ± 0.32	11.75 ± 0.80 *
Phos (mM)	1.55 ± 0.46	1.52 ± 0.34
Amylose (U L^−1^)	1512.00 ± 20.47	1610.50 ± 32.24 *

AST: aspartate aminotransferase, ALT: alanine aminotransferase, ALP: alkaline phosphatase, TG: triglycerides, LDL: low-density lipoprotein, CK: creatine kinase, HDL: high density lipoprotein, Na: sodium, K: potassium, Cl: chloride, Lact: lactate, Ca: calcium, Chol: cholesterol, Phos: phosphorus, Trig: triglycerides. * Significantly different from vehicle control at *p* < 0.05.

**Table 5:** Relative rat organ weights after feeding with palm puree for 28 d in a subchronic toxicity study.

Parameters	Dose (mg kg^−1^)			
	0	500	1000	2000
		Male		
Heart	0.37 ± 0.17	0.37 ± 0.07	0.39 ± 0.12	0.40 ± 0.24
Kidney (R)	0.37 ± 0.28	0.38 ± 0.33	0.35 ± 0.11	0.38 ± 0.24
Kidney (L)	0.38 ± 0.26	0.38 ± 0.22	0.43 ± 0.12	0.40 ± 0.36
Liver	3.5 ± 0.57	3.88 ± 0.22	4.03 ± 0.34	4.08 ± 0.69
Spleen	0.30 ± 0.40	0.25 ± 0.13	0.25 ± 0.15	0.23 ± 0.49
Lung	0.58 ± 0.36	0.51 ± 0.13	0.69 ± 0.40 *	0.76 ± 0.57 *
		Female		
Heart	0.43 ± 0.10	0.38 ± 0.04	0.40 ± 0.13	0.37 ± 0.09
Kidney (R)	0.35 ± 0.06	0.31 ± 0.18	0.35 ± 0.07	0.35 ± 0.06
Kidney (L)	0.36 ± 0.06	0.39 ± 0.17	0.35 ± 0.05	0.36 ± 0.11
Liver	4.63 ± 0.98	4.61 ± 0.62	4.43 ± 0.27	4.35 ± 0.56
Spleen	0.27 ± 0.08	0.29 ± 0.17	0.27 ± 0.15	0.29 ± 0.14
Lung	0.57 ± 0.16	0.77 ± 0.38 *	0.74 ± 0.33 *	0.76 ± 0.32 *

* Significantly different from vehicle control at *p* < 0.05.

**Table 6:** Haematology data for male and female rats treated with oral palm puree for 28 d.

Parameter	Dose (mg kg^−1^ d^−1^)			
	0	500	1000	2000
Male				
RBC (× 10^12^ L^−1^)	6.66 ± 0.40	7.83 ± 0.66	6.84 ± 0.96	5.34 ± 0.87
Haemoglobin (g L^−1^)	131.33 ± 0.51	142.40 ± 0.08	127.2 ± 0.99	119.33 ± 0.87
PCV (L L^−1^)	0.40 ± 0.10	0.42 ± 0.46	0.37 ± 0.05	0.34 ± 0.87
MCV (fL)	59.6 ± 0.96	54 ± 0.64	55 ± 0.22	52.6 ± 0.47
MCHC (g L^−1^)	349.75 ± 0.45	339.4 ± 0.82	339.6 ± 0.88	351 ± 0.53
WBC (× 10^9^ L^−1^)	4.99 ± 0.38	4.86 ± 0.88	4.32 ± 0.14	3.58 ± 0.67
Neutrophils (× 10^9^ L^−1^)	0.47 ± 0.20	0.55 ± 0.14 *	0.59 ± 0.23 *	0.38 ± 0.12
Lymphocytes (× 10^9^ L^−1^)	5.08 ± 0.79	6.06 ± 0.50	3.43 ± 0.97 *	3.03 ± 0.89 *
Female				
RBC (× 10^12^ L^−1^)	7.17 ± 0.39	7.38 ± 0.52	6.79 ± 1.01	6.52 ± 0.33
Haemoglobin (g L^−1^)	132.6 ± 0.42	136.6 ± 0.50	128 ± 0.40	121.52 ± 0.48
PCV (L L^−1^)	0.43 ± 0.04	0.44 ± 0.04	0.40 ± 0.04	0.36 ± 0.08
MCV (fL)	59.6 ± 0.51	59.6 ± 0.78	58.8 ± 0.32	57.0 ± 0.45
MCHC (g L^−1^)	311.8 ± 0.89	312.4 ± 0.73	322.8 ± 0.63	336.0 ± 0.06
WBC (× 10^9^ L^−1^)	4.91 ± 0.25	4.19 ± 0.62	4.62 ± 0.71	3.93 ± 0.97
Neutrophils (× 10^9^ L^−1^)	0.39 ± 0.24	0.35 ± 0.06	0.27 ± 0.63	0.30 ± 0.21
Lymphocytes (× 10^9^ L^−1^)	4.19 ± 0.08	3.46 ± 0.53	3.64 ± 0.93	3.29 ± 0.62

RBC: red blood cell count, MCV: mean corpuscular volume, MCH: mean corpuscular haemoglobin, MCHC: mean corpuscular haemoglobin concentration, WBC: white blood cell count, PCV: packed cell volume. * Significantly different from vehicle control at *p* < 0.05.

**Table 7:** Serum biochemical parameters of male and female rats treated with various palm puree doses for 28 d in a subchronic toxicity study. Data are means ± SD for groups of five animals/sex/dose. * (*p* < 0.05) vs. control group.

Parameters	Dose (mg kg^−1^)			
	0	500	1000	2000
Male				
Trig (mM)	1.07 ± 0.18	0.87 ± 0.15 *	0.83 ± 0.25 *	0.87 ± 0.32 *
LDL (mM)	0.55 ± 0.14	0.64 ± 0.1	0.63 ± 0.1	0.51 ± 0.07
HDL (mM)	1.25 ± 0.28	1.12 ± 0.27	1.3 ± 0.1	1.23 ± 0.08
Chol (mM)	2.44 ± 0.16	2.64 ± 0.17	2.47 ± 0.56	2.52 ± 0.51
LDH (mM)	2202.75 ± 25.8	1824.42 ± 31.5 *	1994.61 ± 17.5 *	1838.24 ± 11.7 *
Lact (mM)	5.01 ± 0.64	5.96 ± 3.21	4.91 ± 0.64	5.34 ± 0.78
CK (U L^−1^)	1408.52 ± 14.67	1164.25 ± 23.78 *	1112.23 ± 41.12 *	1319.2 ± 25.61
Total protein (g L^−1^)	54.83 ± 0.62	54.74 ± 0.10	55.46 ± 0.18	54.68 ± 0.82
Albumin (g L^−1^)	36.73 ± 0.45	28.8 ± 0.5060	36.76 ± 0.55	35.64 ± 0.94
Na (mM)	142.25 ± 0.26	142 ± 2.35	143.4 ± 0.89	139.6 ± 0.52
K (mM)	6.03 ± 0.99	5.26 ± 0.38	4.56 ± 0.29 *	4.98 ± 0.39 *
Cl (mM)	101.25 ± 0.96	100.8 ± 0.30	101.6 ± 0.52	99.2 ± 0.64
Uric acid (µM)	66.58 ± 5.42	78.15 ± 3.67	58.93 ± 1.16	56.68 ± 6.0 *
Urea (mM)	7.85 ± 2.25	8.22 ± 1.54	7.14 ± 0.61	8.20 ± 0.39
Creatinine (mM)	56.25 ± 4.27	57.8 ± 6.57	56.0 ± 3.39	57.0 ± 4.30
ALT (mM)	65.7 ± 1.60	83.06 ± 1.47	72.02 ± 1.56	74.68 ± 5.91 *
ALP (U L^−1^)	135.0 ± 8.12	152.2 ± 3.64	146.4 ± 2.68	162.4 ± 7.42
AST (U L^−1^)	200.3 ± 17.53	172.08 ± 13.6	177.9 ± 35.52	186.04 ± 36.37
Glucose (mM)	14.48 ± 2.23	15.62 ± 4.36	14.36 ± 2.44	15.74 ± 6.19
Phos (mM)	3.22 ± 0.32	2.53 ± 0.43	2.3 ± 0.29	2.02 ± 0.71
Amylose (U L^−1^)	1839.50 ± 87.9	2303.80 ± 70.5	2294.0 ± 92.35	2439.6 ± 65.26
Female				
Trig (mM)	1.00 ± 0.32	1.27 ± 0.48	0.90 ± 0.14	0.84 ± 0.19 *
Total protein (g L^−1^)	58.96 ± 0.21	61.3 ± 047	55.48 ± 0.30	55.48 ± 0.30
LDL (mM)	0.32 ± 0.11	0.25 ± 0.07	0.36 ± 0.02	0.37 ± 0.07
HDL (mM)	1.11 ± 0.23	0.89 ± 0.09	0.77 ± 0.19	1.21 ± 0.10
Chol (mM)	2.51 ± 0.38	2.53 ± 0.51	1.84 ± 0.14	2.39 ± 0.46
LDH (mM)	2173.6 ± 81.65	2085.20 ± 74.10	2083 ± 83.74	1982.34 ± 65.57
Lact (mM)	6.27 ± 0.11	6.44 ± 1.01	6.10 ± 1.71	6.76 ± 1.91
CK (U L^−1^)	1163 ± 11.70	1187 ± 12.64	1110.6 ± 12.31	1129.5 ± 16.72
Total protein (g L^−1^)	58.96 ± 1.21	61.3 ± 2.47	56.08 ± 1.18	55.48 ± 2.30
Albumin (g L^−1^)	40.22 ± 5.5	42.26 ± 9.2	39.06 ± 7.91	39.12 ±.4.7
Na (mM)	144.2 ± 0.13	143.4 ± 0.67	144.2 ± 0.84	144.6 ± 0.36
K (mM)	4.48 ± 0.52	5.4 ± 0.76	4.74 ± 0.94	5.28 ± 0.62
Cl (mM)	101.24 ± 1.19	100.65 ± 1.14	103.27 ± 0.84	104.02 ± 2.74
Uric acid (µM)	59.98 ± 0.97	50.16 ± 0.46	57.10 ± 0.91	63.10 ± 2.19
Urea (mM)	7.36 ± 0.61	8.14 ± 0.61	8.44 ± 0.59	8.92 ± 1.33
Creatinine (mM)	57.21 ± 2.68	56.2 ± 3.70	55.6 ± 3.78	58.4 ± 2.30
ALT (mM)	49.97 ± 1.53	49.17 ± 0.20	45.88 ± 7.38	43.74 ± 6.64
ALP (U L^−1^)	120.46 ± 0.36	114.64 ± 2.52	134.22 ± 0.64	141.08 ± 1.66
AST (U L^−1^)	143.71 ± 15.43	150.31 ± 31.62	162.25 ± 52.12	152.67 ± 34.88
Glucose (mM)	11.7 ± 2.50	11.04 ± 0.43	13.32 ± 0.33	13.64 ± 0.52
Phos (mM)	2.25 ± 0.33	2.26 ± 0.22	2.06 ± 0.65	2.02 ± 0.43
Amylose (U L^−1^)	1813.16 ± 85.25	1708.48 ± 73.20	1424.10 ± 87.25	1575.23 ± 69.27

AST: aspartate aminotransferase, ALT: alanine aminotransferase, ALP: alkaline phosphatase, LDL: low-density lipoprotein, CK: creatine kinase, HDL: high density lipoprotein, Na: sodium, K: potassium, Cl: chloride, Lact: lactate, Ca: calcium, Chol: cholesterol, Phos: phosphorus, Trig: triglycerides.
